# Perioperative and Functional Results for Robot-assisted Radical Cystectomy with Totally Intracorporeal Neobladder in Male Patients via the Vesica Patavina (Ves.Pa.) Technique: IDEAL Stage 2a Report

**DOI:** 10.1016/j.euros.2023.09.001

**Published:** 2023-09-22

**Authors:** Fabrizio Dal Moro, Fabio Zattoni, Elisa Tonet, Alessandro Morlacco, Giovanni Betto, Giacomo Novara

**Affiliations:** Urology Clinic, Department of Surgery, Oncology and Gastroenterology, University of Padua, Padua, Italy

## Abstract

**Background:**

Robot-assisted radical cystectomy (RARC) offers several advantages over open surgery, but intracorporeal neobladder construction (INC) is a challenging procedure. The vesica patavina (Ves.Pa.) refinement is a modification of the original technique that simplifies the neobladder configuration and reduces the risk of complications.

**Objective:**

To present a stage 2a IDEAL (Idea, Development, Exploration, Assessment and Long-term follow-up) report on RARC with INC using the Ves.Pa. technique.

**Design, setting, and participants:**

This was a prospective study of consecutive male patients undergoing RARC and Ves.Pa. INC performed by a single surgeon for muscle-invasive or non–muscle-invasive, bacillus Calmette-Guérin–refractory urothelial bladder cancer in a tertiary referral center.

**Surgical procedure:**

RARC with INC using the refined Ves.Pa. technique.

**Measurements:**

Complications were classified using the Clavien-Dindo scheme, and functional outcomes were assessed using validated questionnaires.

**Results and limitations:**

A total of 20 male patients were treated. The median operative time was 382 min, and the median estimated blood loss was 350 ml. The incidence of high-grade complications was extremely low, with only one patient experiencing a grade IIIa complication. All patients had clear surgical margins. At median follow-up of 12 mo, statistically significant differences in all the functional scores measured were observed. Specifically, 6-mo parameters were all significantly worse than at baseline (all *p* < 0.05). No patients required intermittent catheterization. Severe urinary incontinence was experienced by approximately 25% of the patients. The median number of pad used was 0 during the day and 1 at night. The study is limited by its small sample size, single-center design, and short follow-up.

**Conclusions:**

RARC with the refined Ves.Pa. technique for INC is safe, feasible, and replicable. The technique simplifies the procedure and reduces the risk of complications. The study results suggest acceptable oncological and functional outcomes over short-term follow-up.

**Patient summary:**

We report our initial experience with robot-assisted removal of the bladder and construction of a new bladder using our modified technique, called Ves.Pa., in patients with bladder cancer. The technique is simple to perform. We observed a low rate of high-grade complications, and patients had surgical margins negative for cancer in all cases and fair functional outcomes at 12-month follow-up.

## Take Home Message

We report our initial experience with robot-assisted radical cystectomy and intracorporeal neobladder construction via a refined vesica patavina (Ves.Pa) technique in 20 male patients. Surgical outcomes were favorable, with clear surgical margins and acceptable oncological outcomes. Ves.Pa. is easy to perform and has advantages over other neobladder techniques, such as no stapler use and the creation of a spherical shape using a unique ileal configuration.

## 1 Introduction

Radical cystectomy (RC) with pelvic lymphadenectomy is the standard of care for both high-risk non–muscle-invasive bladder cancer and muscle-invasive disease [1], [2]. With the diffusion of robotic surgery, robot-assisted RC (RARC) is increasing in popularity as an alternative to open or pure laparoscopic surgery because of appealing perioperative and functional outcomes [3], [4].

Following either open RC or RARC, most patients receive an ileal conduit for urinary diversion [5], [6]. Over the decades, we have developed considerable experience with orthotopic neobladders in our center. In the era of open surgery, we reported an original technique for construction of an ileal neobladder, called vescica ileale Padovana (V.I.P.) [7], which has been extensively used in our center [8] and many other centers worldwide. Although a total intracorporeal robotic configuration of the V.I.P. technique has been reported [9], [10] and several other neobladder techniques have been adapted for the robotic surgery setting [11], we elected to modify the original V.I.P. technique for a robotic approach, and proposed the vesica patavina (Ves.Pa.) technique. The pouch was developed in an ex vivo model and is an intracorporeal orthotopic neobladder with exclusively intracorporeal suturing that respects the essential principles of ideal diversions [12]. Specifically, we decided to simplify the original V.I.P. design by removing the funnel configuration of the bladder neck to reduce the risk of clean intermittent catheterization, which was quite high in our experience with the V.I.P. [8]. Moreover, taking advantages of barbed sutures, we opted for a completely hand-sewn neobladder, avoiding the use of staplers, which could increase the risk of stone formation in long-term survivors.

According to the IDEAL (Idea, Development, Exploration, Assessment and Long-term follow-up) model for safe surgical innovation, our proposal represents stage 0 of the framework [13]. The technique was adopted by Whelan et al. [14], who reported on the first three cases performed, representing stage 1 of the IDEAL framework.

Since the original description of the Ves.Pa. technique, we have implemented a minor modification that excludes the non-detubularized 5-cm proximal segment used in the original protocol. The purpose of the present paper is to report the refined surgical Ves.Pa. technique and its results in a series of men with bladder cancer undergoing RARC and neobladder construction (stage 2a of the IDEAL framework).

## 2 Patients and methods

### 2.1 Study setting

From May 2021 to December 2022, RARC and intracorporeal neobladder construction (Ves.Pa. pouch) were performed by a single surgeon in 20 consecutive male patients. The indication for RARC was muscle-invasive or non–muscle-invasive, bacillus Calmette-Guérin (BCG)-refractory urothelial bladder cancer in all the cases, in accordance with the European Association of Urology (EAU) guidelines [1], [2]. The exclusion criteria were age >75 yr, Charlson comorbidity index >2, and the presence of clinically locally advanced or metastatic disease. All the patients underwent chest and abdominal computed tomography scans for preoperative staging.

No bowel preparation was performed. Our protocol for early recovery after surgery includes early mobilization, oralization, and gastrointestinal stimulation with chewing gum [15]. Antibiotic prophylaxis with a third-generation cephalosporin was used in all patients. Venous thromboembolism prophylaxis with low-molecular-weight heparin for 30 d and elastocompressive stockings until hospital discharge was used in all cases, as recommended by the EAU guidelines [16]. A nonopioid perioperative pain management protocol was used in all patients.

### 2.2 Surgical technique

**Fig. 1:**
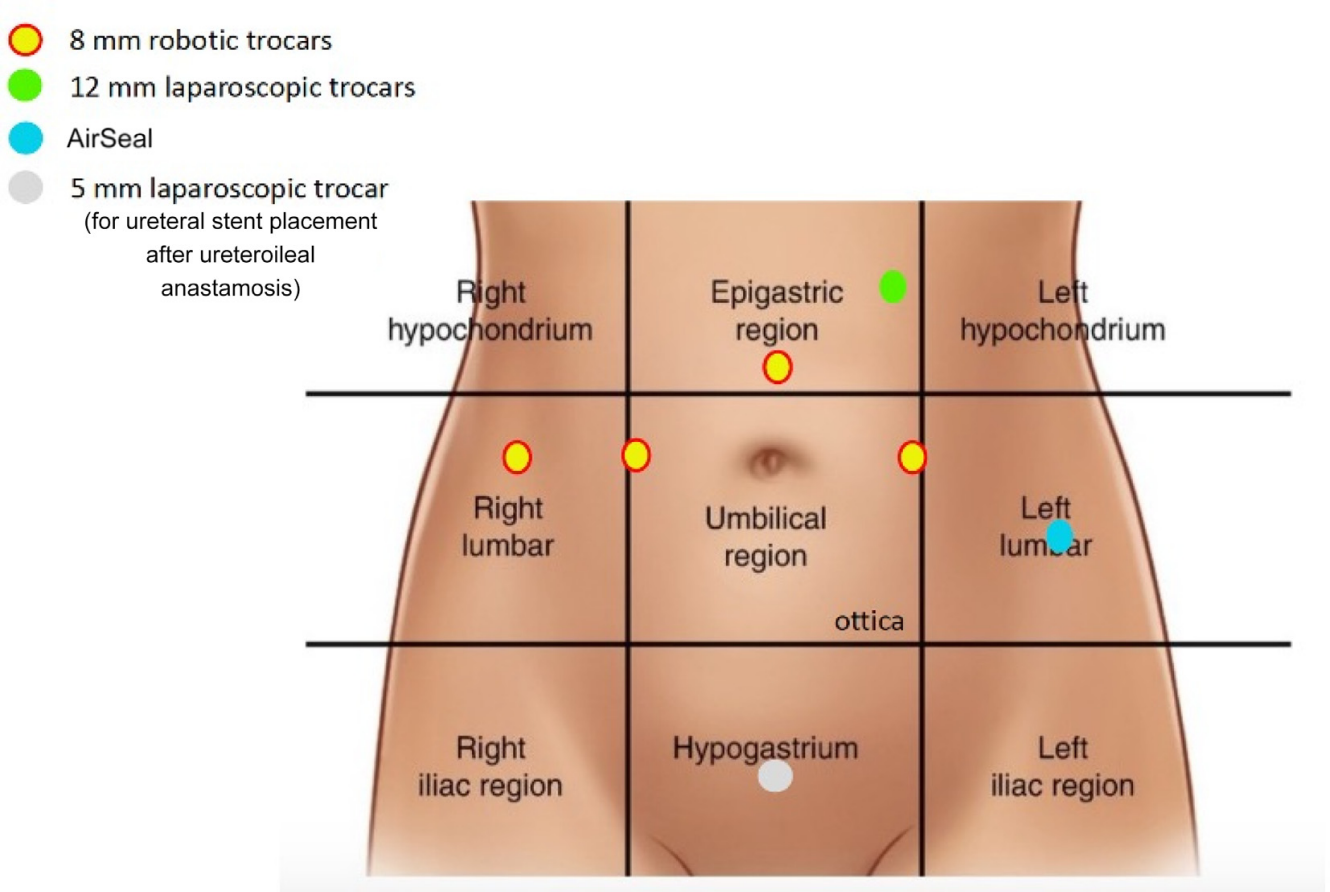
Trocar placement.

All the procedures were performed using a da Vinci Si or X system. The patient was placed in a head-down 29° Trendelenburg position. Foam-cushion table liners were used to prevent the patient from sliding in this position. The legs were in stirrups with minimal hip flexion. The knees were flexed a gentle 30°, and the legs were spread to accommodate the robotic surgical system. A four-arm configuration was adopted in all the cases, with the fourth arm in the right iliac fossa. All the procedures used one vessel sealer, one tip-up fenestrated grasper, one monopolar scissors, and one needle driver, together with a 30° scope. An AirSeal flow system was used in all cases, placed in the left iliac fossa. A 12-mm assistant port was placed between arms 1 and 2. A further 5-mm trocar was placed in the hypogastrium at the time of ureter reimplantation to introduce the ureteral catheters (Fig. 1).

RARC and lymph node dissection (common, external, internal iliac, obturator, and presacral nodes) were performed as previously reported [17] following all the surgical principles for RC suggested by the Pasadena consensus meeting [18], [19].

Special attention was reserved for preserving a flap of the visceral peritoneum, which is sutured distally to the periurethral tissue using double-needle 3-0 Filbloc sutures (Assut Europe, Magliano dei Marsi, Italy) for hemostasis in the cystectomy bed and to provide support for the posterior wall of the neobladder.

In comparison to the original Ves.Pa. procedure [12], the refined technique uses a 40-cm ileal loop that is entirely detubularized, without the closed 5-cm proximal segment used in the original protocol.

#### 2.2.1 Choice of ileal segment and urethroileal anastomosis

As performed for many intracorporeal robotic neobladders, the initial step for neobladder construction is identification of the most sloped part of the ileum at a minimum distance of 20 cm from the ileocecal valve. Once the ileal loop is approximated to the urethral stump, the first step for neobladder configuration is anastomosis between the ileal loop and the urethral stump using an 18 Ch Foley catheter. The anastomosis is performed via the Van-Velthoven technique with a double-needle 3-0 Filbloc suture (5/8 needles). If needed, additional sutures with 3-0 Monosyn using a HR-26 needle (B Braun, Melsungen, Germany) are inserted to reinforce the anastomosis.

#### 2.2.2 Identification of a 40-cm ileal loop, resection, and re-establishment of intestinal continuity

**Fig. 2:**
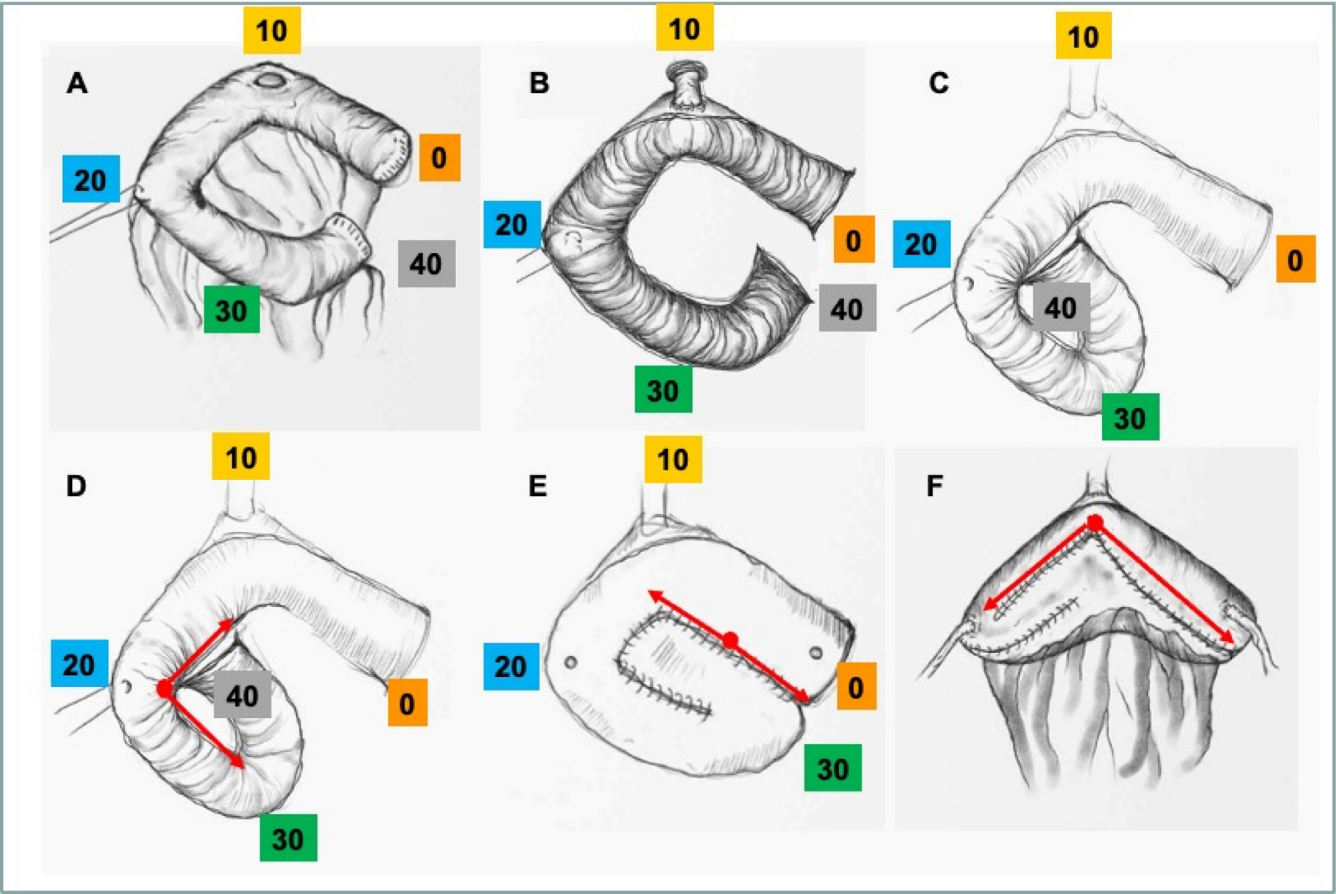
Schematic representation of the vesica Patavina (Ve.Spa.) configuration. (A) Identification of the ileal loop and location of the different marks. (B) Detubularization of the ileal loop at the antimesenteric border after urethroileal anastomosis. (C) First ileal folding matching the medial margins at the 40-cm and 20-cm marks. (D) First semicontinuous suture with Filbloc running cranially towards the 30-cm mark, and the second semicontinuous suture running caudally towards the 10-cm mark to complete the first folding. (E) Second folding matching the medial margins at the 30-cm and 0-cm marks, and two semicontinuous sutures, starting in the middle of the suture line and running cranially towards the 0-cm mark and caudally towards the 10-cm mark. (F) Final folding to close the neobladder using two Filbloc semicontinuous sutures, starting in the median line and running towards the ureter reimplantations bilaterally.

**Fig. 3:**
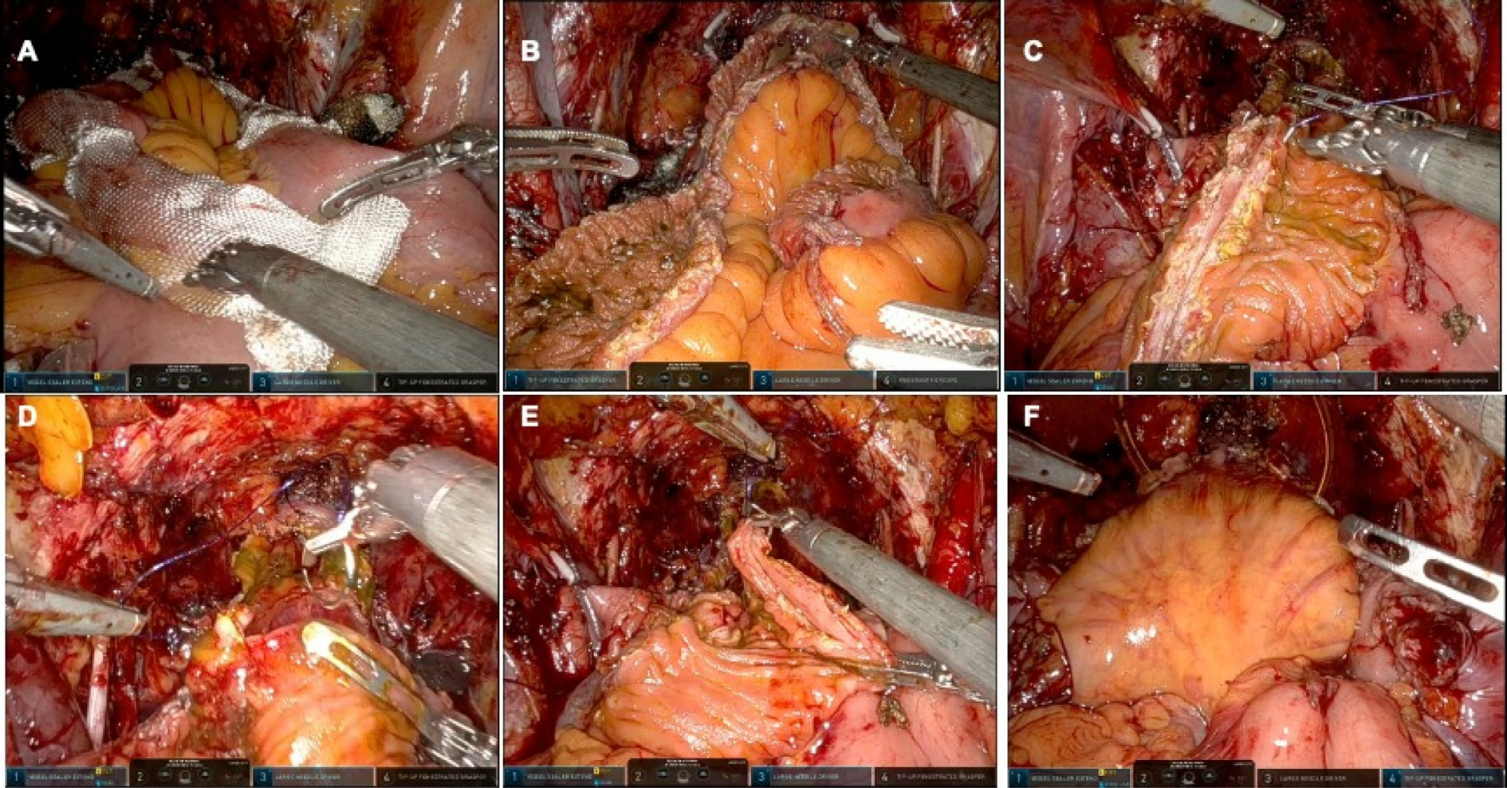
Intraoperative images of the most critical surgical steps in the vesica Patavina (Ves.Pa.) reconfiguration. (A) Identification of a 40-cm ileal loop using TABOTAMP strips. (B) Detubularization of the ileal loop at the antimesenteric border. (C) First ileal folding matching the medial margins at the 40-cm and 200cm marks, and first Filbloc suture running cranially towards the 30-cm mark. (D) Second suture running caudally towards the 10-cm mark to complete the first folding. (E) Second folding matching the medial margins at the 30-cm and 0-cm marks. (F) Final shape of the neobladder after the third ileal folding.

Once the anastomosis is completed, three TABOTAMP strips (Ethicon, Raritan, NJ, USA) are used to identify the correct ileal length. Two 10-cm-long strips are placed proximally and distally to the urethroileal anastomosis to identify the first 20 cm of the ileal loop. Then a 3-0 Vicryl (Ethicon) stay suture is inserted (SH-1 Plus needle, Ethicon) at the 20-cm mark. A 20-cm-long TABOTAMP strip marks the last 20 cm of the ileal loop cranially (Figs. 2A and 3A). Then the ileal resection and laterolateral ileal anastomosis are performed with ECHELON FLEX 60 ENDOPATH staplers (Ethicon) in standard fashion.

#### 2.2.3 Detubularization and neobladder configuration

The 40-cm isolated ileal loop is detubularized along the antimesenteric border (Figs. 2B and 3B). Then the first ileal folding is performed, matching the medial margins at the 40-cm and 20-cm marks (Figs. 2C and 3C). Double-needle 3-0 Filbloc is used for two semicontinuous sutures, one running cranially towards the 30-cm mark and one running caudally towards the 10-cm mark (Figs. 2D and 3D). Next, the second folding is performed, matching the medial margins at the 30-cm and 0-cm marks. Double-needle 3-0 Filbloc is again used for two semicontinuous sutures, starting in the middle of the suture line and running cranially towards the 0-cm mark and caudally towards the 10-cm mark (Figs. 2E and 3E).

#### 2.2.4 Ureteral reimplantation

At this point, the ureters are directly anastomosed to the neobladder on their respective sides over 6 Ch ureteral catheters using three semicontinuous sutures with 3-0 Monosyn and a HR-26 needle.

#### 2.2.5 Final folding of the neobladder

The final folding is performed to close the neobladder using double-needle 3-0 Filbloc for semicontinuous sutures, starting in the median line and running towards the ureter reimplantation (Fig. 2F). Finally, the ends of these last two sutures are used to anchor the remnants of the parietal peritoneum to the neobladder so that the ureteric reimplantation is located extraperitoneally. A 10-mm Jackson-Pratt drain is left in place at the end of the procedure.

### 2.3 Follow-up

Patients were discharged home after complete canalization and regular diet restart, with ureteral catheters and urethral catheter in place. After 2–3 wk, a scan was performed on an outpatient basis; in cases with no evidence of extravasation of contrast medium, the ureteral catheters and urethral catheter were removed one by one in the outpatient clinic. Patients were then reassessed after 1 mo and every 3 mo thereafter, or more frequently if clinically indicated by signs or symptoms, in the outpatient clinic for oncological and functional follow-up.

### 2.4 Data collection

All data were collected prospectively by medical staff. Preoperative data collected included age, gender, body mass index, American Society of Anesthesiologists score, Charlson comorbidity index, prior pelvic surgery, prior bladder cancer therapies, and neoadjuvant chemotherapy administration. Perioperative data comprised operative time, estimated blood loss, perioperative transfusion rate, intraoperative complications, total number of lymph nodes removed, length of hospital stay, 30-d and 90-d postoperative complications, and the readmission rate. Intraoperative and postoperative complications were reported according to the Satava and Clavien-Dindo classification [20], [21]. The present report is in accordance with the EAU recommendations for reporting of complications [22]. Oncological outcomes recorded were urothelial (urethral and ureteral) and soft-tissue positive margins, histology, pathological tumor stage (TNM), positive lymph nodes, and clinical recurrence. Local and distal recurrences were defined as clinical relapse in soft tissue or invasion of lymph nodes, confirmed on imaging.

Italian-validated translations of the American Urological Association Symptom Index (AUA-SI) [23], International Consultation on Incontinence Questionnaire-Urinary Incontinence Short Form (ICIQ-UI SF) [24], and International Index of Erectile Function [25] questionnaires were administered at baseline and at 6-mo and 12-mo follow-up. Patients were also asked how many pads they were using during the day and at night. The severity of urinary incontinence was graded according to the scheme proposed by Klovning et al. [26] using ICIQ-UI SF scores: 0 = fully continent; 1–5 = slight incontinence; 6–12 = moderate incontinence; 13–18 = severe incontinence; and 19–21 = very severe incontinence. All procedures were in accordance with the ethical standards established in Italy.

### 2.5 Statistical analysis

Continuous variables are reported as the median and interquartile range (IQR). Categorical variables are reported as the frequency and percentage. A Wilcoxon test was used to assess the change over time for continuous variables. All statistical analyses were performed with SPSS for Macintosh v28.0 (IBM Corp., Armonk, NY, USA).

## 3 Results

Table 1 summarizes the characteristics of the 20 patients. Eleven patients underwent surgery because of high-grade, non–muscle-invasive urothelial cancer, which was refractory to BCG in most cases. Neoadjuvant chemotherapy was received by 40% of the ten patients with muscle-invasive disease.

**Table 1:** Characteristics of the 20 male patients who underwent robot-assisted radical cystectomy and intracorporeal neobladder construction via the Ve.Spa. technique

Parameter	Result
Median age at surgery, yr (IQR)	65 (53–67)
Median body mass index, kg/m^2^ (IQR)	27 (25–30)
Median CCI (IQR)	0 (0–0.7)
Median age-adjusted CCI (IQR)	2 (1–2)
Prior abdominal surgery, *n* (%)	5 (25)
Appendectomy	2 (10)
Laparoscopic cholecystectomy	2 (10)
Open left hemicolectomy	1 (5)
ASA class II or III, *n* (%)	18 (90)
Clinical T stage, *n* (%)	
Tis	2 (10)
Ta	5 (25)
T1	4 (20)
T2	9 (45)
Clinical N+ stage, *n* (%)	3 (15)
High-grade urothelial cancer, *n* (%)	19 (95)
Prior treatment for non–muscle-invasive disease	
Intravesical chemotherapy	1 (10)
Intravesical bacillus Calmette-Guérin	9 (82)
NAC for muscle-invasive disease, *n* (%)	3 (33)

ASA = American Society of Anesthesiologists; CCI = Charlson comorbidity index; IQR = interquartile range; NAC = neoadjuvant chemotherapy

Table 2 summarizes intraoperative and perioperative data. A single intraoperative complication occurred, in a patient who had previously undergone a left hemicolectomy. During the initial laparoscopic adhesiolysis for trocar placement, a minor bowel injury was sutured laparoscopically without further sequelae. The case was completed robotically as scheduled (Satava grade 1).

**Table 2:** Intraoperative and perioperative data

Parameter	Result
Median operating room time, min (IQR)	382 (330–425)
Median estimated blood loss, ml (IQR)	350 (200–575)
Intraoperative transfusion, *n* (%)	2 (10)
Intraoperative complication, *n* (%)	1 (5)
Intensive care admission, *n* (%)	2 (10)
Median nasogastric tube time, d (IQR)	0 (0–1)
Median time to mobilization, d (IQR)	2 (1–2)
Median time to flatus, d (IQR)	2 (1–3)
Median time to stool passage, d (IQR)	4 (3–5)
Median time to a liquid diet, d (IQR)	2 (1–3)
Median time to a free diet, d (IQR)	3 (2–4)

The median length of stay was 7.5 d (IQR 6–9), but seven patients (35%) required readmission to hospital. The urethral catheter was removed after a median of 29 d (IQR 16–36) and the ureteral catheters were removed after a median of 27 d (IQR 15–36).

Table 3 summarizes all the perioperative complications. Overall, 18 complications occurred in nine patients (45%). Within 30 d, three patients (15%) experienced grade 1 and five patients (25%) experienced grade 2 complications. Three patients (15%) experienced complications between postoperative days 30 and 90, of which two (10%) were grade 2 and one (5%) was a grade 3 complication. The only grade 3 complication was a case of drain entrapment, which required a 5-cm laparotomy for removal. No ureteral complications and no stone disease in the neobladder were observed during follow-up.

**Table 3:** Postoperative complications observed in the 20 patients

Complication	Cases, *n* (%)	Grade
**Up to postoperative day 30**		
Gastrointestinal		
Paralytic ileus^a^	4 (20)	Grade 1 (*n* = 2), grade 2 (*n* = 2)
Diarrhea	1 (5)	Grade 1
Genitourinary		
Lymphocele	1 (5)	Grade 2
Pulmonary		
Pleural effusion	1 (5)	Grade 1
Cardiac		
Atrial fibrillation	1 (5)	Grade 2
Neurologic		
Delirium	1 (5)	Grade 1
Peripheral neuropathy	1 (5)	Grade 1
Infectious		
Pelvic collection	1 (5)	Grade 1
Fever >38°C for >2 d	3 (15)	Grade 2
Deep venous thrombosis	1 (5)	Grade 2
**Postoperative day 30 to day 90**		
Infectious		
Fever >38°C for >2 d	2 (10%)	Grade 2
Other		
Drain entrapment	1 (5%)	Grade 3

a Inability to achieve a bowel movement by postoperative day 5 with no signs of small bowel obstruction.

All the patients had urothelial cancer on RARC pathology, including one micropapillary carcinoma and one plasmacytoid variant. Only three patients (15%) had T3 disease. All surgical margins were negative. The median number of nodes removed was 16 (IQR 12–27). Only a single patient (5%) had lymph node involvement. At median follow-up of 12 mo (IQR 7–17), one patient had experienced local tumor recurrence after neoadjuvant chemotherapy and RARC, with final RARC histology of ypT3bN2 urothelial carcinoma. All the patients were alive at last follow-up.

Table 4 summarizes the functional outcomes over median follow-up of 12 mo (IQR 7–17). During follow-up, significant differences in all the scores measured were observed. Specifically, the 6-mo results were all significantly worse than the baseline values (all *p* < 0.05). Conversely, 12-mo and 6-mo scores were mostly similar. At follow-up, most patients had mild to moderate lower urinary tract symptoms according to the AUA-SI score, with no patient using intermittent catheterization. Most of the patients reported slight or moderate urinary incontinence. The most common conditions during which patients experienced incontinence were sleep, coughing/sneezing, and exercise; Supplementary Table 1 provides details of the data at 6-mo and 12-mo follow-up. The median number of pads used was 0 (IQR 0–2) during the day and 1 (IQR 1–1.5) at night at 6-mo follow-up, and 0 (IQR 0–2) during the day and 1 (IQR 1–1) at night at 12-mo follow-up.

**Table 4:** Functional results in the 20 patients who underwent robot-assisted radical cystectomy and intracorporeal neobladder construction via the Ve.Spa. technique

Parameter	BL	6-mo FU	12-mo FU	*p* value	
				6 mo vs BL	12 mo vs 6 mo
Median AUA-SI score (IQR)	4 (1–8)	15 (8–21)	8 (6–17)	<0.01	<0.05
Median AUA-SI QoL score (IQR)	1 (0–2)	3 (2–4)	2 (1–4)	0.02	0.1
Median ICIQ-UI score (IQR)	0 (0–0)	8 (6–12.5)	7 (6–13)	<0.01	0.6
Median number of pads/d (IQR)	0 (0–0)	0 (0–2)	0 (0–2)	0.03	0.2
Median number of pads/night (IQR)	0 (0–0)	1 (1–1.5)	1 (1–1)	<0.001	0.3

AUA-SI = American Urological Association Symptom Index; BL = baseline; FU = follow-up; ICIQ-UI = International Consultation on Incontinence Questionnaire-Urinary Incontinence; IQR = interquartile range; QoL = quality of life.

## 4 Discussion

We report here our initial experience with RARC and intracorporeal neobladder construction according to a refinement of the Ves.Pa. technique. We observed an extremely low rate of high-grade complications, with clear surgical margins in all cases, acceptable oncological outcomes, and fair functional outcomes at 12-mo follow-up.

Following RC, most patients receive an ileal conduit for urinary diversion [5]. There is conflicting evidence concerning the advantages of a neobladder over an ileal conduit in terms of perioperative outcomes and quality of life [27]. However, it is not infrequent that younger and fit patients prefer a neobladder diversion to preserve their body image. Several surgical techniques for neobladder construction have been reported over the decades, including some novel techniques performed via extracorporeal, intracorporeal, or hybrid robotic approaches. Robotic intracorporeal construction of a neobladder is a demanding surgical procedure with a steep learning curve [28]. However, an intracorporeal robotic approach might be considered preferable over extracorporeal approaches to maximize the benefits of minimally invasive surgery (eg, minimizing evaporative fluid losses from the peritoneum, reducing the skin incision length, allowing more limited ureteric mobilization, and potentially reducing postoperative complications) and some studies have even demonstrated slightly better functional outcomes for robotic procedures [29].

Owing to the lack of long-term functional data for intracorporeal diversions, the choice of surgical technique for intracorporeal diversion is often a matter of personal choice by the surgeon. However, the classic surgical principles of detubularization, double folding, and neobladder configuration should be respected to achieve adequate capacity, compliance, and low pressure [11]. The Ves.Pa. neobladder technique fulfils all these criteria and offers the advantage of being relatively easy to perform. Moreover, it includes only intracorporeal suturing and no use of a stapler for configuration of the neobladder, as in other neobladders [9]. Moreover, the Ves.Pa. neobladder does not have a funnel configuration for the bladder neck, as was the case with the V.I.P. neobladder. This is considered an advantage for voiding symptoms, as it prevents posterior prolapse of the neobladder, which often occurs in the long term. Finally, the ureters are reimplanted on their respective sides, avoiding the need for extensive mobilization of the left ureter and facilitating more extended ureteral resection if needed. For all these reasons, we believe that the refined Ves.Pa. technique may represent an optimal option for intracorporeal neobladder construction.

The present study is important for several reasons. We report here a complete description of the revised Ves.Pa. technique, which corresponds to stage 2a of the IDEAL framework, and detailed data according to the current EAU standard for reporting of complications [19]. Moreover, we used validated questionnaires to report functional outcomes at median follow-up of 12 mo. Overall, we believe that we have provided sufficient details to allow reproduction of the surgical technique, as well as results of good methodological quality.

However, our study is not devoid of limitations. First, the series is small, patients were well selected, and a single surgical team performed all the procedures. All of these factors can impact the external validity of our results and comparisons with other intracorporeal surgical techniques, which should be evaluated in stage 2b studies according to the IDEAL framework. Second, the series includes the team’s learning curve for the procedure. Wijburg et al. [28] reported that among nine high-volume centers, more than 130 cases were needed to reach a plateau in 90-d major complications following RARC with intracorporeal diversions (both conduit and neobladder). Given that we performed 20 RARC procedures with an intracorporeal ileal conduit within the enrolment period, we can hypothesize that our complication rate will further decrease over time. We were not able to report the time to daytime or nighttime continence because functional outcomes in our study were assessed at 6-mo and 12-mo follow-up only. However, the follow-up duration can be considered relatively short, so functional outcomes could improve over time and/or new long-term complications could occur. Finally, we did not use patient diaries or pad tests for an objective assessment of urinary continence, tools that have been used in studies comparing functional outcomes after open or robotic neobladder construction with a variety of different continence definitions [29], [30]. However, we do believe that validated questionnaires such as the ICIQ-UI SF are a solid tool for capturing and grading urinary continence.

## 5 Conclusions

We report our initial experience with RARC and intracorporeal neobladder construction according to the refined Ves.Pa. technique. Our technique offers the advantages of being easy to perform and includes only intracorporeal suturing, with no use of a stapler for neobladder configuration. Moreover, it does not involve a funnel configuration for the bladder neck and the ureters are reimplanted on their respective sides. We observed an extremely low rate of high-grade complications, with clear surgical margins in all cases, acceptable oncological outcomes, and fair functional outcomes at 12-mo follow-up.

  ***Author contributions:*** Giacomo Novara had full access to all the data in the study and takes responsibility for the integrity of the data and the accuracy of the data analysis.

  *Study concept and design*: Dal Moro, Novara.

*Acquisition of data*: Tonet, Betto.

*Analysis and interpretation of data*: Zattoni, Novara.

*Drafting of the manuscript*: Zattoni, Novara.

*Critical revision of the manuscript for important intellectual content*: Dal Moro, Morlacco, Betto.

*Statistical analysis*: Novara.

*Obtaining funding*: None.

*Administrative, technical, or material support*: None.

*Supervision*: Dal Moro.

*Other*: None.

  ***Financial disclosures:*** Giacomo Novara certifies that all conflicts of interest, including specific financial interests and relationships and affiliations relevant to the subject matter or materials discussed in the manuscript (eg, employment/affiliation, grants or funding, consultancies, honoraria, stock ownership or options, expert testimony, royalties, or patents filed, received, or pending), are the following: None.

  ***Funding/Support and role of the sponsor:*** None.
