# Supplementary material for: Perioperative and Functional Results for Robot-assisted Radical Cystectomy with Totally Intracorporeal Neobladder in Male Patients via the Vesica Patavina (Ves.Pa.) Technique: IDEAL Stage 2a Report
Source: Eur Urol Open Sci. 2023 Sep 22;57:8–15. doi: 10.1016/j.euros.2023.09.001 (PMC10522971; doi:10.1016/j.euros.2023.09.001)
Supplement: Supplementary Table 1 [file mmc1.docx]

**Supplementary Table 1: further detail of lower urinary tract symptoms and urinary incontinence at baseline, 6-, and 12-mo follow-up.**

| **Variables** | **Baseline** | **6-mo follow-up** | **12-mo follow-up** |
| --- | --- | --- | --- |
| Lower urinary tract symptoms severity according to AUA-SI  - Mild (score ≤ 7)  - Moderate (score 8 – 19)  - Severe (score ≥ 20) | 15 (75%)  5 (25%)  - | 3 (15%)  12 (60%)  5 (25%) | 7 (35%)  10 (50%)  3 (15%) |
| Urinary incontinence severity according to ICIQ-UI   - Slight (score 1 – 5) - Moderate (score 6 – 12) - Severe (score 13 – 18) | -  -  - | 3 (15%)  12 (60%)  5 (25%) | 4 (20%)  10 (50%)  6 (30%) |
| ICIQ-UI Short form – item 6: when does urine leak  -  before you can get to the toilet  - cough or sneeze  - when asleep  - when physically active/exercising  - all the time | -  -  -  -  - | 4(20%)  6 (30%)  14 (70%)  9 (45%)  3 (15%) | 4 (20%)  6 (30%)  11 (55%)  7 (35%)  4 (20%) |
| Pad per day  - 0 pad  - 1 pad  - >1 pad | 100%  -  - | 10 (50%)  5 (25%)  5 (25%) | 10 (50%)  4 (20%)  6 (30%) |
| Pad per night  - 0 pad  - 1 pad  - >1 pad | 100%  -  - | 2 (10%)  14 (70%)  34 (20%) | 4 (20%)  15 (75%)  1 (5%) |
